# Physiology of Menstruation

**Published:** 1859-09

**Authors:** Thos. S. Denny

**Affiliations:** Atlanta, Ga.


### ARTICLE III.

*Physiology of Menstruation.*—By Thos. S. Denny, M. D., Atlanta, Ga.

Not with a view of opposing the theory of the non-secretion of the menstrual fluid, but for the purpose of having difficulties between the two theories cleared up, I put together the few following thoughts and queries, and solicit explanations from any members of the profession disposed to consider them:

Medicines calculated to restore the secretions of other organs are very commonly employed to bring on the flow when absent. Alteratives proper, as well as other means, the warm bath, &c., &c., are frequently resorted to with success. The smallest discharge brought on during the most violent sufferings, will afford relief and restore the patient from the utmost anxiety and a state of high febrile excitement to a perfect calm, as if quieted by a powerful but innocuous anodyne. Venœsection alone will frequently bring about this highly favorable state of things. This would seem as if the secretion being brought to its healthy condition, however small the quantity—the organ being restored to its true function—the rest of the system was prepared to work in harmony.

Very brisk catharsis will also frequently bring on the discharge in a plethoric habit. It may act as a stimulant, the action being sympathetic with the alimentary canal, or by draining off a determination, and relieving a congestion, or in some other way.

The restoration of the healthy secretion of the liver in Dysentery by alteratives, relieves all the distressing symptoms previously existing, and this favorable change generally terminates the disease, unless extremely complicated with other distinct disorders calculated to keep up a morbid irritation in the system. We know the fact that the favorable action, or effect of calomel is often manifested by the *sensations* not very long after its reception, and a considerable time before any action on the bowels, or any other proof of its alterative powers. I have been personally cognizant of a favorable change in the secretion of that organ, by that means, and I have seen the same in others.

It is claimed by a recent contributor to your Journal that "During the menstrual flow in the female * * * the principal organs of generation are furnished with an extra amount of blood," &c. I am acquainted with the history of a case in this city, (not likely to be an isolated one,) of a married lady, in whom the catamenia had not made their appearance for several months; her health remained very good, however, and she became pregnant, but remained ignorant of her situation, in consequence of the non-appearance, until she was suddenly apprised of it by the motion of the child. She was delivered of a perfectly healthy and fully developed child, at (we have no reason to imagine otherwise,) the end of the usual term of gestation. Here, the uterus and ovaria must have been in a healthy condition for conception; but where was the previous "extra amount of blood," which "is converted into tissue" after impregnation? If the secretion was absent in the above case so was also the "extra amount of blood." Did her conception bring on the needed supply? I am willing to allow that it did, in preference to believing it to have had the effect of inducing a condition favorable to secretion. If the discharge be a secretion, we can readily imagine many causes, *especially cold*, well adapted to suppress it; if only the result of a high state of excitement, may not that condition be supposed, *a priori*, capable of resisting causes regarded as likely to produce disorder?

Then, there is the opposite state of things, a continuation of the flow, monthly, for a limited time, and, occasionally, during the whole period of pregnancy. Are these as satisfactorily accounted for under the new as the old theory? Are we to suppose that, under the former, there is more blood than is needed to go to the increase in growth of the uterus, and that nature continues to throw off the superabundance?

I am well aware of having advanced nothing to elucidate the subject, and hope to be enlightened hereafter.

